# Fibrin-associated diffuse large B-cell lymphoma in a hemorrhagic cranial arachnoid cyst

**DOI:** 10.1186/s40478-017-0463-3

**Published:** 2017-08-07

**Authors:** Daniel Kirschenbaum, Peter Prömmel, Flavio Vasella, Eugenia Haralambieva, Ewerton Marques Maggio, Robert Reisch, Marc Beer, Ulrike Camenisch, Elisabeth J. Rushing

**Affiliations:** 1 Institute of Neuropathology, University Hospital of Zurich, Zurich, Switzerland; 2 Center for endoscopic and minimalinvasive Neurosurgery, Clinic Hirslanden, Zurich, Switzerland; 3 Department of Pathology and Molecular Pathology, University Hospital of Zurich, Zurich, Switzerland

**Keywords:** Diffuse large cell B-cell lymphoma, Fibrin, Pathology, Neurosurgery, Arachnoid cyst

Arachnoid cysts are a common incidental finding on magnetic resonance imaging (MRI) performed for other clinical reasons. They can be found in the brain or spine and are mostly of congenital origin due to splitting of the arachnoid membrane. The vast majority are asymptomatic, with signs and symptoms varying according to size and location. Intracystic hemorrhage is a rare complication. In symptomatic cases, treatment predominantly consists of endoscopic fenestration [4, 5, 7].

In recent years, the features of diffuse large cell B-cell lymphoma (DLBCL), referred to as fibrin-associated DLBCL, have been reported in cases of chronic blood effusions [1]. Fibrin-associated DLBL, which has a favorable clinical outcome, should be distinguished from chronic inflammation-associated DLBCL, which is an aggressive tumor. Fibrin-associated DLBCL has been described in cases throughout the body; however, only isolated intracranial cases have been described, which were found in the subdural space. Here we present the case of an elderly man with an unsuspected fibrin-associated DLBCL in an arachnoid cyst. We would like to draw attention to this entity, which has likely been underestimated in the routine evaluation of subdural hematoma (SDH) or subarachnoid cysts.

An 81-year-old man presented with intermittent tremor and gait ataxia. The patient was diagnosed with classical parkinsonism and L-Dopa treatment was started. Later, the patient developed short-term memory disturbances and the gait ataxia progressed. Based on the MRI findings, a right frontotemporal arachnoid cyst with focal bleeding was suspected. Intraoperatively, the lesion presented as an arachnoid cyst filled with thick, whitish fluid reminiscent of empyema. The cyst was washed out and the lining of the cyst was resected (Fig. 1). The postoperative course of the patient was uneventful with good recovery of the neurological status. After the pathological diagnosis was rendered, a whole-body PET-CT showed no other lesions. Due to the relatively advanced age of the patient, therapy with rituximab and lenalidomide was initiated.

**Fig. 1:**
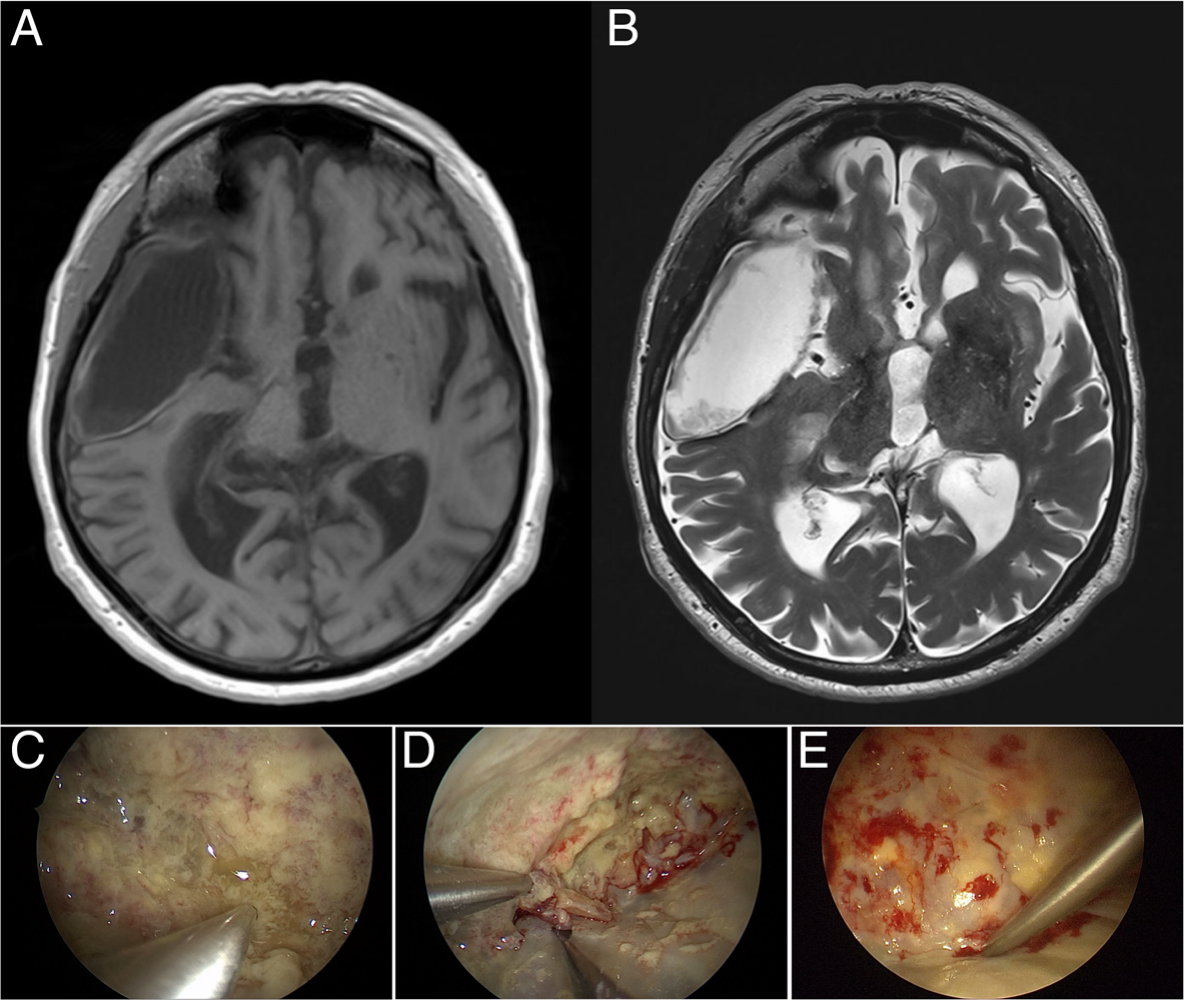
T2-weighted MRI with right temporal arachnoid cyst with signs of intracystic hemorrhage (**a**). Postoperative control-MRI shows total resection of the tumoral tissue within the cyst (**b**). Initial intraoperative endoscopy showed a thick, yellowish lining of the inner wall of the cyst, (**c**) which was removed under endoscopy (**d**) with eventual total resection (**e**)

Hematoxylin-eosin-stained sections (Fig. 2a) revealed small, discohesive islands of large atypical cells against a background of abundant fibrin, without evidence of a large mass-forming lesion. On immunohistochemistry, the atypical cells were strongly CD20 (Fig. 2b) positive. The cells showed high proliferative activity with multiple mitotic figures and a Mib-1 proliferation index of over 80% (Fig. 2c). Immunohistochemical preparations were strongly positive for CD30, Bcl2 (Fig. 2d) and IRF-4 (nuclear, Fig. 2e), with only focal positivity for BCL6. In addition, CD5 immunolabeling was detected in scattered non-atypical cells, which were small reactive T-cells in contrast to the large atypical cells. CD10, TdT, pancytokeratin and melanocytic markers were negative. In situ hybridization showed the presence of non-coding Epstein-Barr virus (EBV)-associated RNA in the majority of the atypical cells (Fig. 2f). C-MYC immunohistochemistry showed expression in more than 50% of the cells. No *C-MYC*- rearrangement was detected by fluorescent in situ hybridization. A PCR based analysis revealed a monoclonal rearrangement in the *IgH* Gene.

**Fig. 2:**
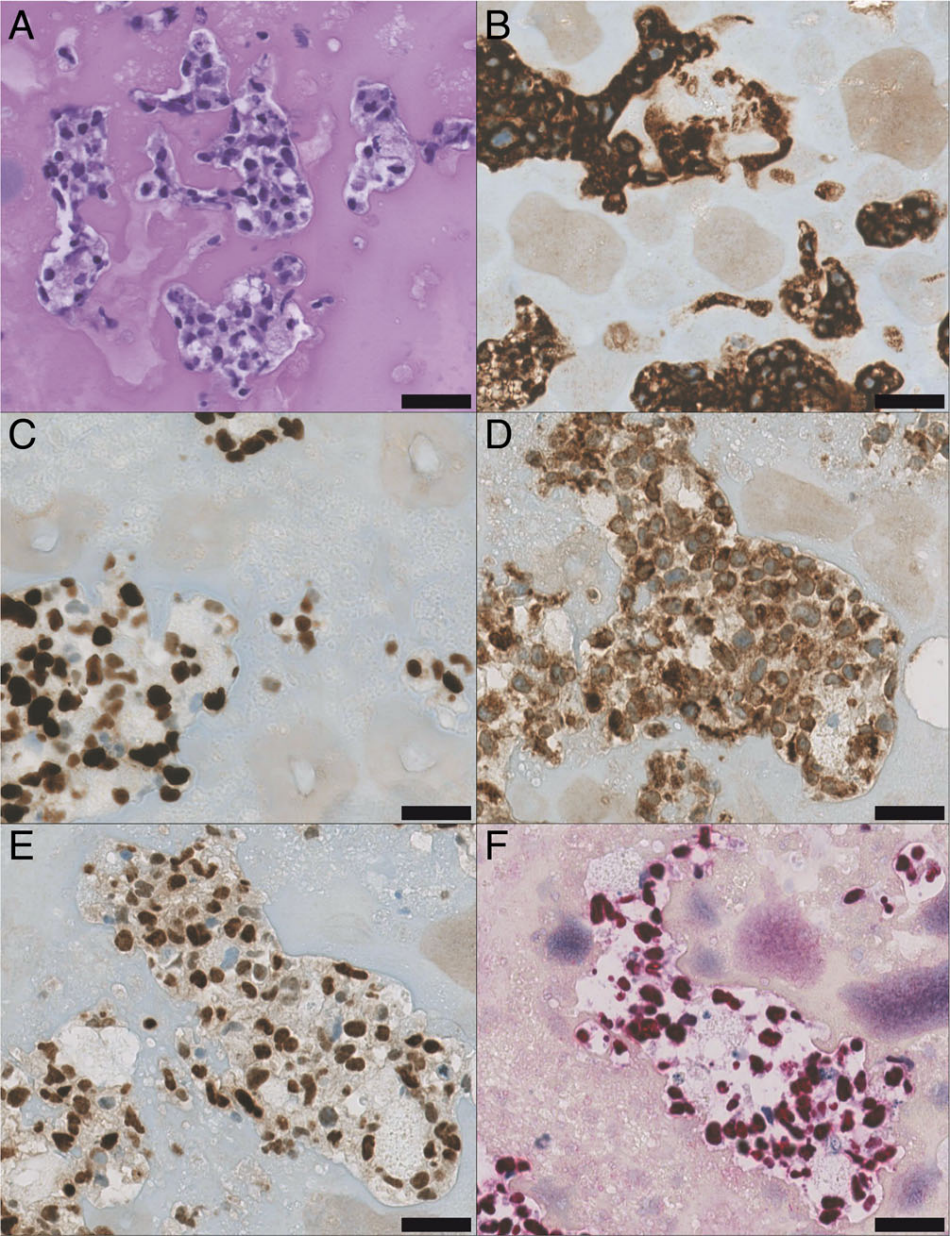
Microscopic pathology. Islands of atypical blastic lymphoid cells are embedded in abundant fibrin (**a**). The cells are diffusely positive for CD20 (**b**), highly proliferative in the Mib-1 (**c**) stain and show strong positivity for BCL2 (**d**), IRF-4 (**e**) and EBV-associated RNA (**f**). Scale bars represent 30 μm

Taken together the diagnosis of a fibrin associated EBV-positive large-cell B-cell lymphoma was rendered.

Diffuse large-cell B-cell lymphomas comprise a group of relatively common hematological malignancies. DLBCL may be associated with chronic inflammation, now considered a rare EBV-associated subtype in immunocompetent individuals. For the most part, DLBL with chronic inflammation has an unfavorable prognosis. EBV-positive DLBL have also been reported as an incidental finding in the setting of chronic hematomas, atrial myxomas and pseudocysts [1]. The median age is 55.5 years with a male: female ratio of 3:1. All cases have been incidental findings, were EBV associated and had an invariably favorable prognosis. Although these lesions have been described in distinct locations, the histology is remarkably similar, with atypical B-cells embedded in a fibrinous background. As reviewed by Boyer et al., three cases of fibrin-associated DLBL have been documented in SDH found in patients with a median age of 66.5 years and a male: female ratio of 4:0. An additional new case was reported by Boyer et al. All except one of these cases showed similar histology, prognosis and an EBV-association [1, 2, 6]. One of the cases was considered a primary lymphoma presenting as a chronic subdural hematoma [2]. We report for the first time a fibrin-associated DLBL in an arachnoid cyst with hemorrhage. In rare cases, arachnoid cysts may harbor either primary or metastatic tumors [3]. In the present case, the diagnosis of an arachnoid cyst was based on radiological and intraoperative assessment. Microscopically, there was no evidence of an arachnoid membrane, which could represent a sampling error. Although the radiographic and intraoperative appearance was virtually pathognomonic of an arachnoid cyst, the microscopic appearance could also be interpreted as a resorbed SDH with recurrent bleeding. In most cases, surgery appears to be curative. In the current case, however, rituximab and lenalidomide therapy was administered. Greater awareness is important in order to more accurately assess the natural history of this entity.

## Abbreviations

DLBCL: Diffuse large-cell B-cell lymphoma
EBV: Epstein-Barr virus
MRI: Magnetic resonance imaging
SDH: Subdural hematoma
